# Structural modification of resveratrol analogue exhibits anticancer activity against lung cancer stem cells via suppression of Akt signaling pathway

**DOI:** 10.1186/s12906-023-04016-6

**Published:** 2023-06-03

**Authors:** Sunisa Thongsom, Satapat Racha, Korrakod Petsri, Zin Zin Ei, Kittichate Visuttijai, Sohsuke Moriue, Masashi Yokoya, Pithi Chanvorachote

**Affiliations:** 1 Center of Excellence in Cancer Cell and Molecular Biology, Faculty of Pharmaceutical Sciences, Chulalongkorn University, Bangkok, 10330 Thailand; 2 Department of Pharmacology and Physiology, Faculty of Pharmaceutical Sciences, Chulalongkorn University, Bangkok, 10330 Thailand; 3 Interdisciplinary Program in Pharmacology, Graduate School, Chulalongkorn University, Bangkok, 10330 Thailand; 4 Department of Laboratory Medicine, Institute of Biomedicine, University of Gothenburg, Gothenburg, 405 30 Sweden; 5 Department of Pharmaceutical Chemistry, Meiji Pharmaceutical University, 2-522-1 Noshio, Kiyose, Tokyo, 204-8588 Japan

**Keywords:** Moscatilin, Resveratrol, Lung cancer, Akt pathway, CSCs, Molecular docking, Molecular dynamics simulations

## Abstract

**Background:**

Compound with cancer stem cell (CSC)-suppressing activity is promising for the improvement of lung cancer clinical outcomes. Toward this goal, we discovered the CSC-targeting activity of resveratrol (RES) analog moscatilin (MOS). With slight structural modification from RES, MOS shows dominant cytotoxicity and CSC-suppressive effect.

**Methods:**

Three human lung cancer cell lines, namely H23, H292, and A549, were used to compare the effects of RES and MOS. Cell viability and apoptosis were determined by the MTT assay and Hoechst33342/PI double staining. Anti-proliferative activity was determined by colony formation assay and cell cycle analysis. Intracellular reactive oxygen species (ROS) were measured by fluorescence microscopy using DCFH_2_-DA staining. CSC-rich populations of A549 cells were generated, and CSC markers, and Akt signaling were determined by Western blot analysis and immunofluorescence. Molecular docking and molecular dynamics (MD) simulations were used to predict the possible binding of the compound to Akt protein.

**Results:**

In this study, we evaluated the effects of RES and MOS on lung cancer and its anti-CSC potential. Compared with RES, its analog MOS more effectively inhibited cell viability, colony formation, and induced apoptosis in all lung cancer cell lines (H23, H292, and A549). We further investigated the anti-CSC effects on A549 CSC-rich populations and cancer adherent cells (A549 and H23). MOS possesses the ability to suppress CSC-like phenotype of lung cancer cells more potent than RES. Both MOS and RES repressed lung CSCs by inhibiting the viability, proliferation, and lung CSC-related marker CD133. However, only MOS inhibits the CSC marker CD133 in both CSC-rich population and adherent cells. Mechanistically, MOS exerted its anti-CSC effects by inhibiting Akt and consequently restored the activation of glycogen synthase kinase 3β (GSK-3β) and decreased the pluripotent transcription factors (Sox2 and c-Myc). Thus, MOS inhibits CSC-like properties through the repression of the Akt/GSK-3β/c-Myc pathway. Moreover, the superior inhibitory effects of MOS compared to RES were associated with the improved activation of various mechanism, such as cell cycle arrest at G2/M phase, production of ROS-mediated apoptosis, and inhibition of Akt activation. Notably, the computational analysis confirmed the strong interaction between MOS and Akt protein. MD simulations revealed that the binding between MOS and Akt1 was more stable than RES, with MM/GBSA binding free energy of − 32.8245 kcal/mol at its allosteric site. In addition, MOS interacts with Trp80 and Tyr272, which was a key residue in allosteric inhibitor binding and can potentially alter Akt activity.

**Conclusions:**

Knowledge about the effect of MOS as a CSC-targeting compound and its interaction with Akt is important for the development of drugs for the treatment of CSC-driven cancer including lung cancer.

**Supplementary Information:**

The online version contains supplementary material available at 10.1186/s12906-023-04016-6.

## Background

Despite advances in early diagnostics, effective drugs, and surgical, and radiotherapy improvement, lung cancer remains the major cause of cancer-related mortality [1, 2]. Failures of lung cancer therapy and the development of metastatic tumors were found to involve cancer stem cells (CSCs), a specific cancer cell subpopulation possessing stem cell properties and tumorigenic potential [3–5]. Recent research has pointed CSCs as promising targets for cancer treatment [6]. For the molecular signaling approach, CSC properties were linked with intracellular stem cell-associated signals, and drugs inhibiting such CSC signals can benefit the treatment of CSC-driving cancers [7].

Currently, CSC-suppressing compounds isolated from plants have been reported, and natural product-derived compounds have potential to be further developed for CSC-targeted therapy [8–10]. Resveratrol (trans-3,5,4′-trihydroxystilbene, RES), a natural polyphenolic compound with potent antioxidant activity, is one such compound that may be useful for CSC suppression [11]. In addition, the anticancer activities of RES have been well established in various cancer types, such as reducing growth rate, inhibiting the production of new blood vessels, and suppressing metastasis [12, 13]. However, RES has limited water solubility and poor bioavailability [14]. Moscatilin (4,4′-dihydroxy-3,3′,5′-trimethoxybibenzyl, MOS) belongs to polyphenolic compounds isolated from *Dendrobium moschatum*, which is a natural trimethoxylated RES analog [15]. Structural modification of RES by the replacement of hydroxyl with methoxy could improve the anticancer activity of RES [16]. Moreover, the modification of the hydroxyl group on the phenol ring of stilbenes can improve bioavailability, and methylation of the hydroxyl group can inhibit rapid metabolism and increase lipophilicity [17]. MOS is hypothesized to improve bioactivities and increase bioavailability compared with RES. Recently, MOS has been shown to suppress the growth of several cancer cells and exert cytotoxic effects more potent than its parent compound RES [18]. However, the therapeutic effect and anticancer mechanism of MOS against lung CSCs compared with RES have not been clarified before.

Pluripotent transcription factors, including Oct4, Nanog, Sox2, and c-Myc, regulate, and maintain CSCs and tumorigenicity [19]. Protein kinase B or Akt was suggested to be important for CSC properties in lung cancers [20]. Akt can phosphorylate Sox2 at the position of T116 and increase Sox2 stability [21]. In addition, c-Myc stability was found to be controlled by Akt activity [22]. Hence, Akt inhibitor would be a promising strategy for CSC suppression [23].

Here, the anticancer effects of RES and its analog (MOS) were compared using both lung cancer adherent and stem-like cells. The abilities of the two similar compounds to suppress growth, induce apoptosis, and suppress CSC phenotypes were evaluated and compared in lung cancer cell lines. Furthermore, we evaluated the effects of RES and MOS on the modulation of the key molecular targets in Akt and their interactions using molecular docking and MD simulations.

## Methods

### Reagents and antibodies

Roswell Park Memorial Institute (RPMI) 1640 medium, Dulbecco’s Modified Eagle’s Medium (DMEM), L-glutamine, and Antibiotic-Antimycotic (100X) (Anti-Anti) were obtained from Gibco (Grand Island, NY, USA). Fetal bovine serum (FBS), phosphate-buffered saline (PBS) and 0.25% trypsin-EDTA were purchased from HyClone (Logan, UT, USA). RES, 3-(4,5-dimethylthiazol-2-yl)-2,5-diphenyltetrazolium bromide (MTT), dimethyl sulfoxide (DMSO), crystal violet, paraformaldehyde, 2′,7′-Dichlorofluorescin diacetate (DCFH_2_-DA), *N*-acetyl-L-cysteine (NAC), RNase A, Hoechst 33342 and propidium iodide (PI) were purchased from Sigma-Aldrich (St. Louis, MO, USA). Radioimmunoprecipitation assay (RIPA) lysis buffer and Immobilon Western Chemiluminescent HRP Substrate was purchased from Millipore (Billerica, MA, USA) and protease inhibitor cocktail was purchased from Roche Molecular Biochemicals (Indianapolis, IN, USA). The primary antibody against CD133 (ab19898) was purchased from Abcam (Cambridge, MA, USA). Sox2 (#3579), phospho-Akt (p-Akt (Ser473), #4060), Akt (#9272), phospho-GSK-3β (p-GSK-3β (Ser9), #9332), GSK-3β (#9832), c-Myc (#5605), β-actin (#4970), and the secondary antibody anti-rabbit IgG (#7074) or anti-mouse IgG (#7076) were provided by Cell Signaling Technology (Danvers, MA, USA).

### Synthesis of MOS

MOS (**SM_7**)

A solution of **5** (50.0 mg, 0.104 mmol) in Tetrahydrofuran (THF) (5.6 mL) was hydrogenated over 10% Pd/C (55% water, 22.2 mg) at room temperature (RT) for 20 h. The catalyst was removed by celite filtration and the filtrate was concentrated in vacuo to give MOS (**SM_7**) (33.6 mg, quant) as a colorless solid.

^1^ H NMR (400 MHz, CDCl_3_) δ: 6.84 (1 H, d, *J* = 7.8 Hz), 6.68 (1 H, dd, *J* = 1.7, 7.8 Hz), 6.61 (1 H, d, *J* = 1.7 Hz), 6.34 (2 H, s), 5.49 (1 H, brs), 5.39 (1 H, brs), 3.84 (6 H, s), 3.84 (3 H, s), 2.81 (4 H, s). ^13^ C NMR (100 MHz, CDCl_3_) δ: 146.8, 146.2, 143.7, 133.6, 132.8, 132.8, 121.0, 114.1, 111.2, 105.1, 56.2, 55.8, 38.4, 37.9. IR (KBr cm^− 1^): 3537, 3010, 2939, 1614, 1515, 1463, 1428, 1325, 1220, 1149, 1114, 1034, 927, 770, 662, 566, 557, 541, 518, 512, 500, 486, 457, 532, 426, 420, 412, 407. EI-MS *m/z* (%): 274 (M^+^, 32), 138 (11), 137 (100). HRMS (EI): Calcd for C_17_H_29_O_5_, 304.1311; Found: *m/z* 304.1307.

4-(benzyloxy)-3-methoxybenzaldehyde (**1**)

A solution vanilline (10.0 g, 65.7 mmol) in CH_3_CN (54 mL) was added NaHCO_3_ (6.29 g, 74.9 mmol, 1.14 equiv.) and KI (1.09 g, 6.57 mmol, 0.1 equiv.), and the obtained solution was heated to 60 °C. After benzyl chloride (8.00 mL, 69.5 mmol, 1.06 equiv.) was added to this solution, refluxed for 5 h. After cooling to RT, the reaction mixture was evaporated under vacuum. The residue was diluted with HCl solution (2.1 mL, 1 mol/L) and extracted with EtOAc (50 mL×3), washed with brine, dried over anhydrous Na_2_SO_4_, and concentrated. The crude product was purified over SiO_2_ column (n-Hex. : EtOAc = 7 : 3) to give **1** (8.33 g, 52%) as a colorless solid.

^1^ H NMR (300 MHz, CDCl_3_) δ: 9.84 (1 H, s), 7.30–7.45 (7 H, m), 6.99 (1 H, d, *J* = 8.2 Hz), 5.25 (2 H, s), 3.95 (3 H, s).

(4-(benzyloxy)-3-methoxyphenyl)methanol (**2**)

A solution of 1 (3.00 g, 12.4 mmol) in methanol (30 mL), tetrahydrofuran [(THF) 30 mL], and H_2_O (3 mL) was added NaBH_4_ (515 mg, 13.6 mmol, 1.1 equiv.) at 0 °C, and the reaction mixture was stirred for 1 h. The reaction was diluted with Et_2_O (30 mL) and quenched with HCl solution (11 mL, 1 mol/L). The obtained solution was evaporated under vacuum. The residue was diluted with H_2_O (10 mL) and extracted with EtOAc (60 mL×3), washed with brine, dried over anhydrous Na_2_SO_4_, and concentrated to give **2** (2.99 g, 100%) as a colorless solid.

^1^ H NMR (400 MHz, CDCl_3_) δ: 7.43 (2 H, d, *J* = 7.1 Hz), 7.36 (2 H, t, *J* = 7.1 Hz), 7.30 (1 H, t, *J* = 7.1 Hz), 6.95 (1 H, d, *J* = 1.6 Hz), 6.86 (1 H, d, *J* = 8.4 Hz), 6.82 (1 H, dd, *J* = 1.6, 8.4 Hz), 5.16 (2 H, s), 4.61 (2 H, s), 3.91 (3 H, s).

diethyl (4-(benzyloxy)-3-methoxybenzyl)phosphonate (**3**)

A solution of NBS (7.65 g, 43.0 mmol, 3.5 equiv.) in CH_2_Cl_2_ (44 mL) was added dimethylsulfide (3.77 mL, 51.6 mmol, 4.2 equiv.) at 0 °C over 7 min. The reaction mixture was stirred at this temperature for 10 min. A solution of **2** (3.00 g, 12.3 mmol) in CH_2_Cl_2_ (44 mL) was cooled at − 18 °C and was added above solution. The reaction mixture was stirred at − 18 °C for 3 h. The reaction mixture was warmed to 0 °C and diluted with H_2_O and extracted with CH_2_Cl_2_ (80 mL×3), washed with saturated NaHCO_3_ solution and H_2_O, dried over anhydrous Na_2_SO_4_, and concentrated. The crude product was dissolved in triethyl phosphite (2.79 mL, 16.1 mmol, 1.24 equiv.). The reaction mixture was stirred at 140 °C for 4 h. After cooling to RT, the reaction mixture was evaporated under vacuum. The residue was purified over SiO_2_ column (n-Hex. : EtOAc = 1 : 9) to give **3** (2.01 g, 45%) as a yellow oil.

^1^ H NMR (400 MHz, CDCl_3_) δ: 7.29–7.44 (5 H, m), 6.89 (1 H, s), 6.82 (1 H, d, *J* = 8.0 Hz), 6.75 (1 H, d, *J* = 8.0 Hz), 5.13 (2 H, s), 3.93–4.11 (4 H, m), 3.89 (3 H, s), 3.04 (2 H, d, *J* = 21.2 Hz), 1.20 (6 H, t, *J* = 7.1 Hz).

4-(benzyloxy)-3,5-dimethoxybenzaldehyde (**4**)

A solution syringaldehyde (10.0 g, 54.9 mmol) in CH_3_OH (33 mL) was added K_2_CO_3_ (9.1 g, 65.9 mmol, 1.2 equiv.) and benzyl bromide (7.82 mL, 65.9 mmol, 1.2 equiv.), and the obtained solution was refluxed for 20 h. After cooling to RT, the reaction mixture was filtered, and the obtained filtrate was evaporated under vacuum. The residue was diluted with H_2_O and extracted with CHCl_3_ (50 mL×3), washed with brine, dried over anhydrous Na_2_SO_4_, and concentrated. The crude product was purified over SiO_2_ column (n-Hex. : EtOAc = 7 : 3) to give **4** (9.40 g, 63%) as a yellow oil.

^1^ H NMR (400 MHz, CDCl_3_) δ: 9.86 (1 H, s), 7.47 (2 H, dd, J = 1.6, 7.3 Hz), 7.28–7.38 (3 H, m), 7.11 (2 H, s), 5.13 (2 H, s), 3.90 (6 H, s).

(*E*)-2-(benzyloxy)-5-(4-(benzyloxy)-3-methoxystyryl)-1,3-dimethoxybenzene (**5**)

A solution of **3** (400 mg, 1.10 mmol, 1.2 equiv.) in THF (5.5 mL) was stirred at − 78 °C and added *t*-BuOK solution in THF (1.46 mL, 1.46 mmol, 1.6 equiv., 1.0 M) over 30 min. The reaction mixture was stirred for 20 min. at the same temperature, and was added **4** (249 mg, 0.915 mmol) in THF (1.0 mL) over 20 min. and the mixture was stirred for 1 h at − 78 °C and for 10 min. at 0 °C. Then, the reaction mixture was stirred for 2 h at RT. The reaction mixture was cooled to 0 °C and diluted with saturated NH_4_Cl solution and extracted with EtOAc (60 mL×3), washed with saturated NH_4_Cl solution and H_2_O, dried over anhydrous Na_2_SO_4_, and concentrated. The crude product was purified over SiO_2_ column (CH_2_Cl_2_) to give **5** (231 mg, 52%) as a colorless solid.

^1^ H NMR (400 MHz, CDCl_3_) δ: 7.49 (2 H, d, *J* = 6.8 Hz), 7.44 (2 H, d, *J* = 7.3 Hz), 7.27–7.39 (6 H, m), 7.07 (1 H, d, *J* = 2.0 Hz), 6.97 (1 H, dd, *J* = 2.0, 8.3 Hz), 6.94 (1 H, d, *J* = 16.1 Hz), 6.88 (1 H, d, *J* = 16.1 Hz), 6.86 (1 H, d, *J* = 8.3 Hz), 6.70 (2 H, s), 5.17 (2 H, s), 5.02 (2 H, s), 3.95 (3 H, s), 3.87 (6 H, s). ^13^ C NMR (100 MHz, CDCl_3_) δ: 153.6, 149.8, 148.0, 137.8, 137.0, 136.6, 133.3, 130.8, 128.5, 128.5, 128.1, 127.8, 127.8, 127.2, 127.0, 119.6, 114.0, 109.3, 103.4, 75.1, 71.0, 56.1, 56.0. IR (KBr cm^− 1^): 3547, 3019, 2399, 1507, 1331, 1214, 1030, 928, 753, 668, 501, 476, 454, 441, 435, 429, 407, 401. EI-MS *m/z* (%): 482 (M^+^, 13), 392 (28), 391 (100), 91 (62). HRMS (EI): Calcd for C_31_H_30_O_5_, 482.2093; Found: *m/z* 482.2092.

### Cell culture

Human lung cancer H23, H292, and A549 cells were purchased from the American Type Culture Collection (Manassas, VA, USA). H23 and H292 cells were cultured in RPMI medium (Gibco). A549 cells were cultured in DMEM medium (Gibco). The medium was supplemented with 10% FBS (HyClone), 2 mM L-glutamine (Gibco), and 1X Anti-Anti (Gibco). Cells with 70 − 80% confluence were trypsinized with 0.25% trypsin–EDTA (HyClone) and subcultured in the same media. The cells were maintained at 37 °C in an incubator with a humidified atmosphere containing 5% CO_2_.

### Preparation of compounds solution

The stock solutions of RES and MOS was prepared at concentration of 50 and 12.5mM in DMSO (Sigma) and stored in aliquots at − 20 °C. RES and MOS with designated final concentrations (0 − 200 µM) were diluted with cell culture media for subsequence experiments with a maximal DMSO concentration less than 0.5% DMSO. DMSO was used as the vehicle control.

### Cell viability assay

The effect of RES and MOS on cell viability in lung cancer cells was assessed by using MTT assay. Briefly, lung cancer cell lines (H23, H292, and A549) were seeded overnight at a density of 1 × 10^4^ cells per well in 96-well plates. The cells were then treated for 24 h with different concentrations (0 − 200 µM) of RES or MOS. After desired incubation, MTT solution (0.5 mg/mL) was added and the cells were incubated in dark for 3 h at 37 °C in the incubator containing 5% CO_2_. The MTT solution was replaced with DMSO (100 µL/well) to dissolve the purple formazan crystal. The absorbance was measured at 570 nm using a microplate reader (Perkin Elmer, Waltham, MA, USA). Percentage of cell viability in relation to the non-treated control was calculated from the optical density (OD) ratio of treated to non-treated control cells. IC_50_ values were calculated using regression analysis from dose-response curves (GraphPad Prism7 software, San Diego,USA). The cancer selectivity index (SI) was calculated by the following equation: SI = mean IC_50_ against normal cells/mean IC_50_ against cancer cells.

### Colony formation assay

Lung cancer cells (H23, A549, and H292) were plated in triplicate into 6-well plates at 300 cells/well. Following overnight attachment, cells were treated with various concentrations (0 − 25 µM) of RES or MOS for 24 h. After treatment, the medium containing RES or MOS was replaced with the fresh medium. The colonies were allowed to form for 7 days, and the medium was changed every two days. For colony staining, colonies were washed once with PBS, then fixed by adding fixative (methanol: acetic acid (3:1, v/v)) for 5 min and stained with crystal violet (0.05% (w/v)) in 4% paraformaldehyde for 30 min. The excess crystal violet was washed with distilled water several times and let air dry at RT. The colonies were photographed with a digital camera, and the acquired images were analyzed using the ImageJ software (National Institutes of Health (NIH), Bethesda, MD, USA).

### Cell cycle analysis

The cell cycle distribution was determined by flow cytometry. A549 cells were plated into 6-well plates at 1 × 10^5^ cells/well overnight and synchronized by serum deprivation for 48 h before treatment. The cells were treated with RES or MOS at 0, 1, 2.5, and 5 µM for 24 h. After treatment, cells were stained with PI using the methods previously described [24]. Flow cytometry was performed on a Guava easyCyte HT flow cytometer (Merck Millipore, Billerica, MA, USA). The DNA content in sub-G1, G0/G1, S, and G2/M phases of the cell cycle was assessed using the Guava InCyte software (Merck Millipore).

### Measurement of intracellular ROS

The intracellular accumulation of reactive oxygen species (ROS) was assessed by fluorescence microscopy. The lung cancer cell lines (A549 and H23) were seeded in 96-well black plates with clear bottom at 1 × 10^4^ cells/well overnight and treated with various concentrations at 0, 2.5, and 5 µM of RES or MOS for 2 h. After treatment, the cells were stained with 10 µM DCFH_2_-DA (D6883, Sigma-Aldrich) for 30 min at 37 °C in the dark. Subsequently, stained cells were photographed under a fluorescent microscope (Nikon ECLIPSE Ts2, Tokyo, Japan).

### Hoechst 33,342 and PI staining assay

Apoptosis was detected by co-staining with Hoechst 33342 and PI. Nuclear morphology was assessed using the DNA dye Hoechst 33342. The lung cancer cell lines (H23, A549 and H292) were seeded in 96-well plates at 1 × 10^4^ cells/well and treated with various concentrations (0 − 10 µM) of RES or MOS for 24 h. For MOS treatment with NAC, the cells were pretreated with 5 mM NAC for 30 min before being exposed to MOS at 2.5 and 5 µM for 24 h. The cells were stained with Hoechst 33342 (10 µg/mL) and PI (1 µg/mL) for 30 min at 37 °C. Then, the cells were visualized under fluorescent microscope (Nikon ECLIPSE Ts2, Tokyo, Japan) and the percentages of apoptotic cells were determined.

### Western blot analysis

A549 and H23 cells were plated overnight. The cells were treated with MOS at 0, 1, 2.5, and 5 µM for 24 h. Following specific treatments, cells were lysed and prepared for Western blotting as was described previously [24]. Nonspecific binding was blocked with 5% skim milk before incubation with the primary antibody (CD133, Sox2, p-Akt, Akt, p-GSK-3β, GSK-3β, c-Myc, and β-actin) at dilution 1:1,000 for overnight at 4 °C. The appropriate secondary antibody (goat anti-rabbit IgG (HRP) or rabbit anti-mouse IgG (HRP)) was diluted at dilution 1:5,000 and incubated for one hour at RT. The enhance chemiluminescence (Immobilon Western HRP Substrate, Millipore) was used to detect protein bands and analyzed by ImageJ (NIH, Bethesda, MD, USA).

### Spheroid formation assay

To generate CSC-rich population, A549 cells were grown in a 24-well ultralow attachment plates at a density of 1.5 × 10^3^ cells/well in DMEM medium containing 1% FBS for 7 days to form primary spheroids. At day 7, primary spheroids were harvested and resuspended as single cells using 0.25% trypsin–EDTA (HyClone) and seed onto 24-well ultralow attachment plates for 14 days to form secondary spheroids. After 14 days of secondary spheroid development, each secondary spheroid was collected, dissociated into a single spheroid of the same size, and treated with non-cytotoxic concentrations (0 − 2.5 µM) of RES or MOS for 3 days. Phase-contrast images of secondary spheroids were captured (day 0, 1, and 3) after treatment using a phase-contrast microscope (Nikon ECLIPSE Ts2). At day 3, a single spheroid was co-stained with Hoechst 33342 and PI at 37 °C for 15 min and photographed under a fluorescence microscope (Nikon ECLIPSE Ts2).

### Immunofluorescence assay

For staining cell monolayers, A549 and H23 cells were seeded at a density of 1 × 10^4^ cells per well in 96-well plates overnight. The cells were then treated with 5 µM of RES or MOS and incubated for 24 h. Following fixation with 4% paraformaldehyde for 15 min, cells were permeabilized with 0.2% (v/v) Triton X-100 and blocked with 10% (v/v) FBS for 20 min at RT. The cells were incubated overnight at 4 °C in the presence of p-Akt or CD133 primary antibodies at a dilution of 1:100 in 4% FBS. After incubation, Alexa Fluor 488 or Alexa Fluor 594 conjugated with goat anti-rabbit IgG secondary antibody at a ratio of 1:500 in 4% FBS was added and incubated for 1 h at RT in the dark. Cell nuclei were stained with Hoechst 33342 (10 µg/mL) for 15 min at RT and then photographed under a fluorescent microscope (Nikon ECLIPSE Ts2, Tokyo, Japan).

For staining spheroid (3D), A549 cells were allowed to form primary and secondary spheroids as described before. At day 14 of secondary spheroid development, CSC-rich populations were treated with 1 µM of RES or MOS for 24 h, and then the cells were stained following the same procedure as described previously for cell monolayer staining.

### Molecular docking

The binding of investigated compounds to Akt1 was performed through molecular docking. The crystal structure of Akt1 (PDB code: 5KCV) bound to miransertib, an oral allosteric Akt inhibitor [25], was downloaded from the Protein Data Bank (PDB). The integrated “loops/refinement” model of UCSF Chimera (version 1.16) [26] was used to reconstruct in the missing residues. The top-ranked model was selected for further analysis. The 3D structures of the natural compounds were downloaded from the PubChem database [27] and optimized with the XTB (version 6.5.0) program package [28] using the GFN2-xTB method with extreme level [29]. UCSF chimera was used to prepare the ligands and receptors, and AutoDock Vina (version 1.2.3) with the Vina forcefield [30] was used to predict the binding modes of compounds. The docking parameters used in this research were the same as in previous reports [31].

### Molecular dynamics (MD) simulations and free energy calculations

The predicted binding modes of targeted compounds from molecular docking studies were used as initial structures for the molecular dynamics (MD) simulation using the Amber18 and AmberTools19 packages. The protein and compound parameters were used for the FF14SB force field [32] and generalized AMBER force field version 2 (GAFF2) plus AM1-BCC [33, 34]. All complex systems were immersed in a 10 A° truncated octahedral box of pre-equilibrated TIP3P water molecules, and all system charges were neutralized by adding three chloride counterions. The systems were subjected to energy minimizations using the first 2500 steps of the steepest descent (SD) method followed by 2500 steps of the conjugate gradient techniques in the Sander module. Next, NVT equilibration reached 310 K. All hydrogen bond atoms were constrained by the SHAKE method, and the particle mesh Ewald (PME) algorithm was used to treat long-range electrostatic interactions under periodic boundary conditions. Finally, MD simulations were carried out for 100 ns. The stability between the natural compounds and Akt1 was measured by the root mean square deviation (RMSD) using the module implemented in AMBER 18. The Molecular Mechanics Generalized Born Surface Area (MM/GBSA) method [35] using the MMPBSA.py module in AMBER18 calculated the binding free energy for the complex systems [36]. The UCSF ChimeraX (version 1.4) program was used to visualize 3D molecular structures and interactions [37].

### Statistical analysis

All experiments were performed at least three times, and all results are expressed as the mean ± standard deviation. Statistical analyses were performed using GraphPad Prism 7.0 (GraphPad Software, La Jolla, CA, USA). The unpaired t-test was used for the statistical analyses between two groups. P < 0.05 was considered statistically significant.

## Results

### Chemistry

**Fig. 1:**
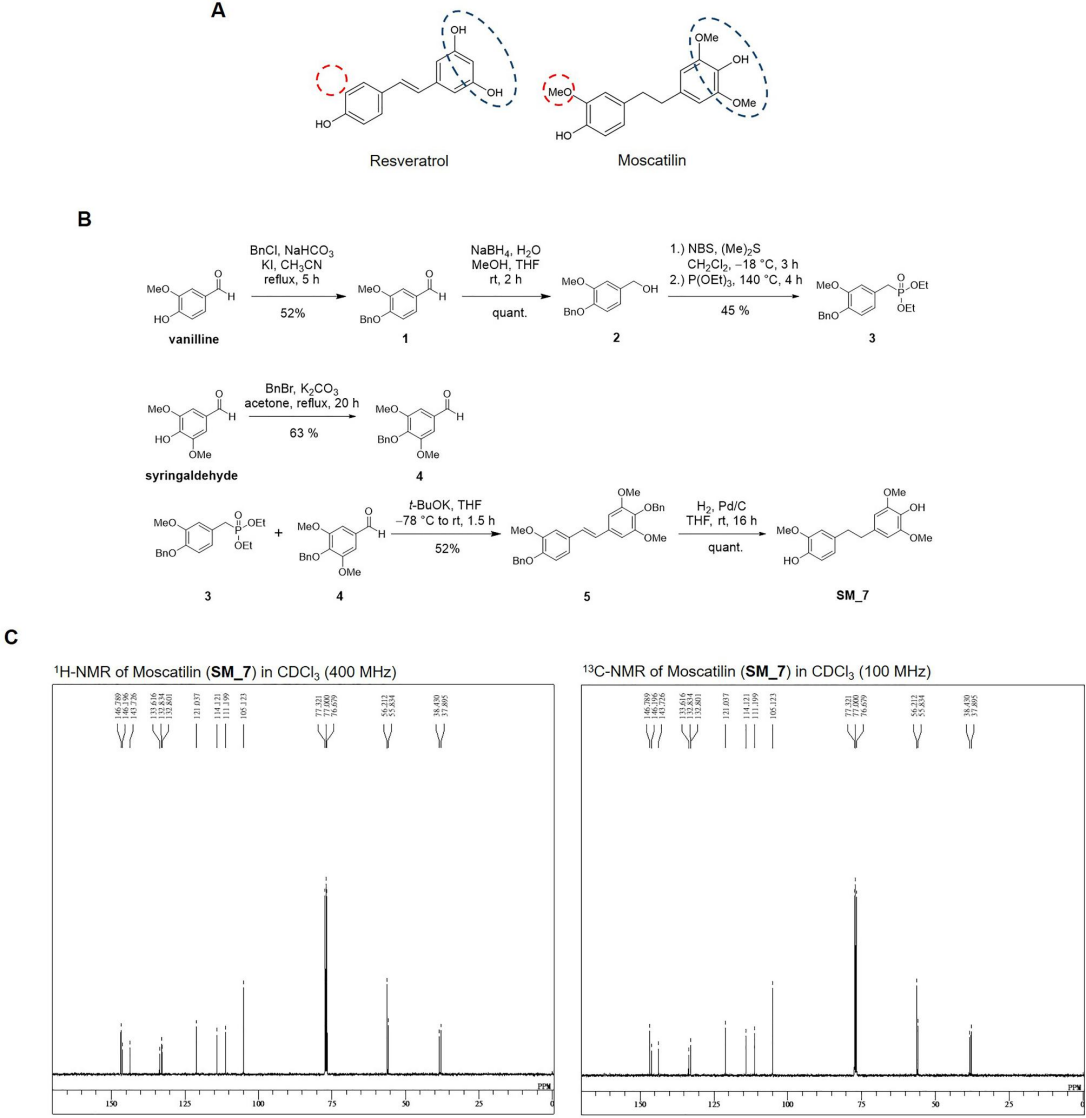
(**A**) Structure Comparison of RES and MOS. (**B**) Synthesis scheme of MOS (SM_7). (**C**) 1 H-NMR and 13 C-NMR analysis of MOS

In this study, we explored the anticancer effects of RES and its analog MOS against human lung cancer cells. The chemical structures of RES and MOS are shown in Fig. 1A. The difference between these two compounds can be detected by referring to differently labeled vertexes, indicating the substitution of the methoxy groups at positions 3, 5, and 4´ and hydroxyl group at position 4. As shown in the synthesis scheme of MOS (**SM_7**) in Fig. 1B, the hydroxy groups of vanilline and syringaldehyde are protected as benzyl ethers **1** and **4**. The aldehyde of **1** was reduced by sodium borohydride to get an alcohol **2**, which was then converted to a bromide followed by the Michaelis–Arbuzov reaction [38] with triethyl phosphite to get **3**, the key reagent for the Horner–Wadsworth–Emmons reaction. The obtained **3** was converted to E-stilbene derivatives **5** by the Horner–Wadsworth–Emmons reaction [39] with aromatic aldehydes **1**. Finally, **5** was catalytically hydrogenated to yield MOS (**SM_7**). ^1^ H and ^13^ C-NMR were identical to that of the natural product [15] (Fig. 1C).

### Comparative effects of RES and MOS on cell viability and induced apoptosis in lung cancer cells

**Fig. 2:**
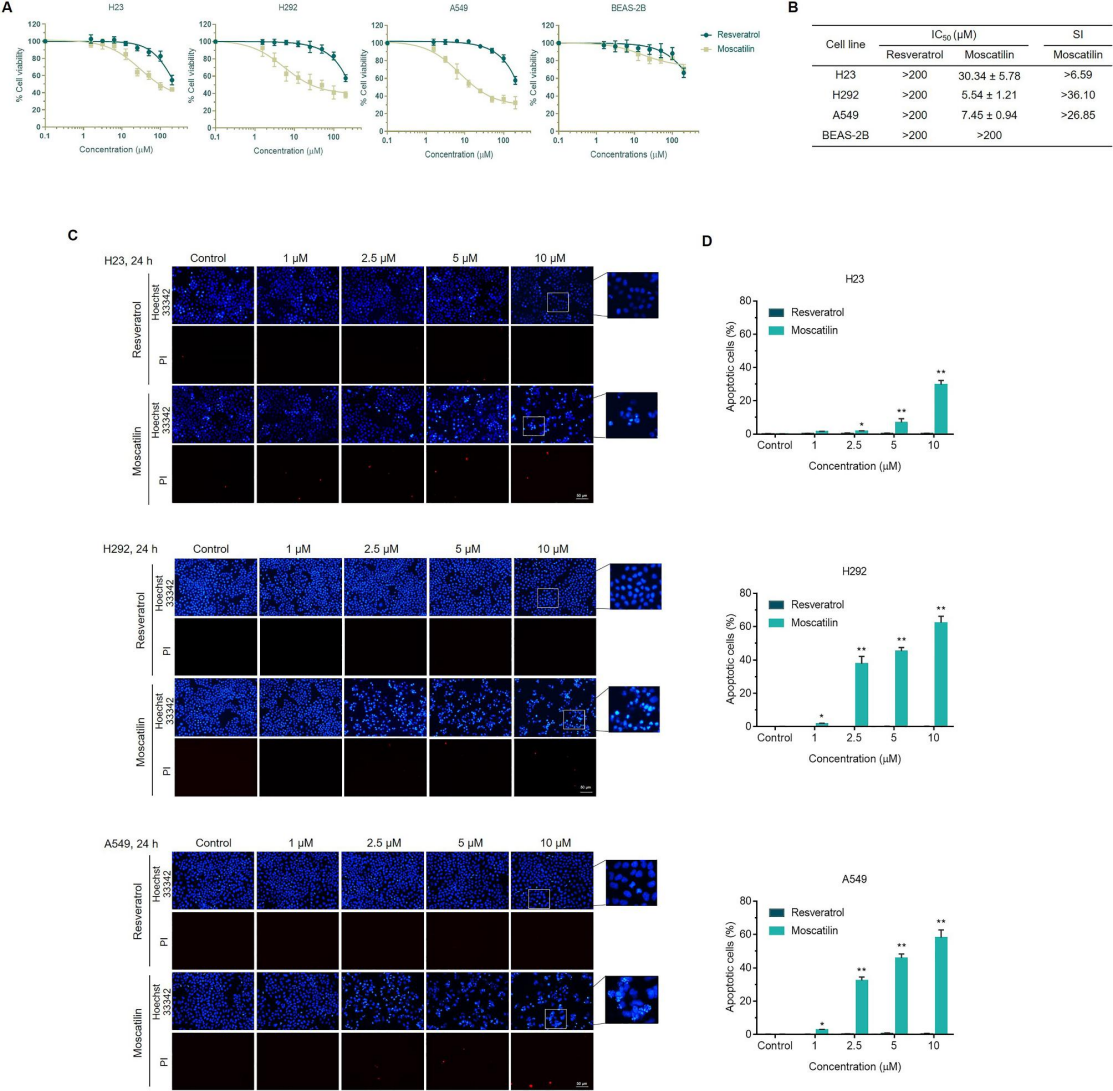
Effects of RES and MOS on cell viability and apoptosis in lung cancer cells (H23, H292, and A549) and normal cells (BEAS-2B). A total of 1 × 10^4^ cells were seeded in 96-well plates and then treated with RES or MOS at the concentrations indicated in the figure for 24 h. (**A**) Cell viability of the cells was determined by MTT assay. (**B**) IC_50_ of three lung cancer cell lines and normal cells after 24 h of RES or MOS treatment. (**C**) The apoptotic cells were evaluated using Hoechst 33342/PI double staining. (**D**) Bar graphs showed the quantitative results of C. Each value is the mean (± SD) from triplicate experiments. *p < 0.05 and **p < 0.01 vs. control

To compare the effects of RES and MOS on anticancer activity, the effects of RES and MOS on cell viability in three lung cancer cell lines (H23, H292, and A549) were evaluated. The cells were treated with various doses (0, 1.5625, 3.125, 6.25, 12.5, 25, 50, 100, and 200 µM) of RES or MOS for 24 h, and cell viability was examined with the MTT assay. Following treatment with MOS, the viability of lung cancer cells was significantly decreased in a dose-dependent manner (Fig. 2A), with a half maximal inhibitory concentration (IC_50_) value of 30.34, 5.54, and 7.45 µM for H23, H292, and A549, respectively, whereas the IC_50_ values of RES were > 200 µM for all lung cancer cell lines (Fig. 2B). The IC_50_ values for MOS were decreased compared with those of RES. In H23 cells, the IC_50_ value of MOS was approximately 8-fold lower than that of RES. H292 and A549 cells appeared to be more sensitive to MOS than H23 cells, whereas the IC_50_ values for MOS were 36- and 27-fold lower than those of RES, respectively. These results suggest that MOS more strongly suppressed the cell proliferation and viability of lung cancer cells than RES. In order to determine whether MOS demonstrates specificity toward lung cancer cells, normal human lung epithelial cells BEAS-2B was also treated with the same range of concentration of MOS to determine its sensitivity. The IC_50_ value for BEAS-2B with treatment of MOS was > 200 µM (Fig. 2A and B). Moreover, the SIs for MOS were > 6.59, > 36.10, and > 26.85 in H23, H292, and A549 cells, respectively (Fig. 2B). A favorable SI more than 1.0 indicates a compound with efficacy against cancer cells greater than the toxicity against normal cells. Regarding RES, since the IC_50_ in both cancer and normal cells were more than 200 µM, the SI could not be calculated. These results indicated that MOS was more selectively cytotoxic to lung cancer cells than normal cells.

Subsequently, we investigated the effects of RES and MOS on the apoptosis of lung cancer cells. The induction of apoptosis by RES and MOS was evaluated in lung cancer cell lines (H23, H292, and A549). The cells were treated with various concentrations (0–10 µM) of RES or MOS for 24 h and then subjected to Hoechst 33342/PI double staining to determine apoptotic and necrotic cells by fluorescence microscope. Hoechst 33342 staining was used to evaluate nuclear condensation and DNA fragmentation, both of which are characteristics of apoptosis [40]. PI fluorescent dye was utilized to identify necrotic or late apoptotic cells, which are distinguished by the loss of plasma and nuclear membrane integrity. As shown in Fig. 2C, MOS induced apoptotic morphological alterations (chromatin condensation and nuclear fragmentation) in all lung cancer cells, whereas RES had no effect. The percentage of apoptotic cells was determined according to the stained image. However, no necrotic cells were detected. As shown in Fig. 2D, MOS dose-dependently increased the percentage of apoptotic cells in all lung cancer cell lines. At maximal concentration (10 µM), the rates of apoptotic cells following treatment with MOS were 29.7%, 62.4%, and 58.2% for H23, H292, and A549, respectively, whereas RES had no effect, with apoptotic cells below 1% for all cancer cells. These results indicated that MOS induces apoptosis in lung cancer cells more effectively than RES, which was consistent with the cell viability assay.

Taken together, our results indicated that MOS had a greater capacity to suppress cell growth and induce apoptosis in lung cancer cells than RES. Structural activity relationship (SAR) is commonly used in drug design and discovery, and it involves modifying the chemical structure of lead compounds to improve their efficacy, potency, selectivity, and safety [41] Thus, structural comparison of these two compounds reveals various chemical differences between RES and MOS, suggesting functional group substitutions alter compound activity. Modification the molecular structure of RES into MOS by the addition of the hydroxyl group at the 3’ position of the phenyl ring and the replacement of hydroxyl of RES with methoxy appeared to enhance the cytotoxicity and induce apoptosis in lung cancer cells.

### MOS increases intracellular ROS in lung cancer cells

**Fig. 3:**
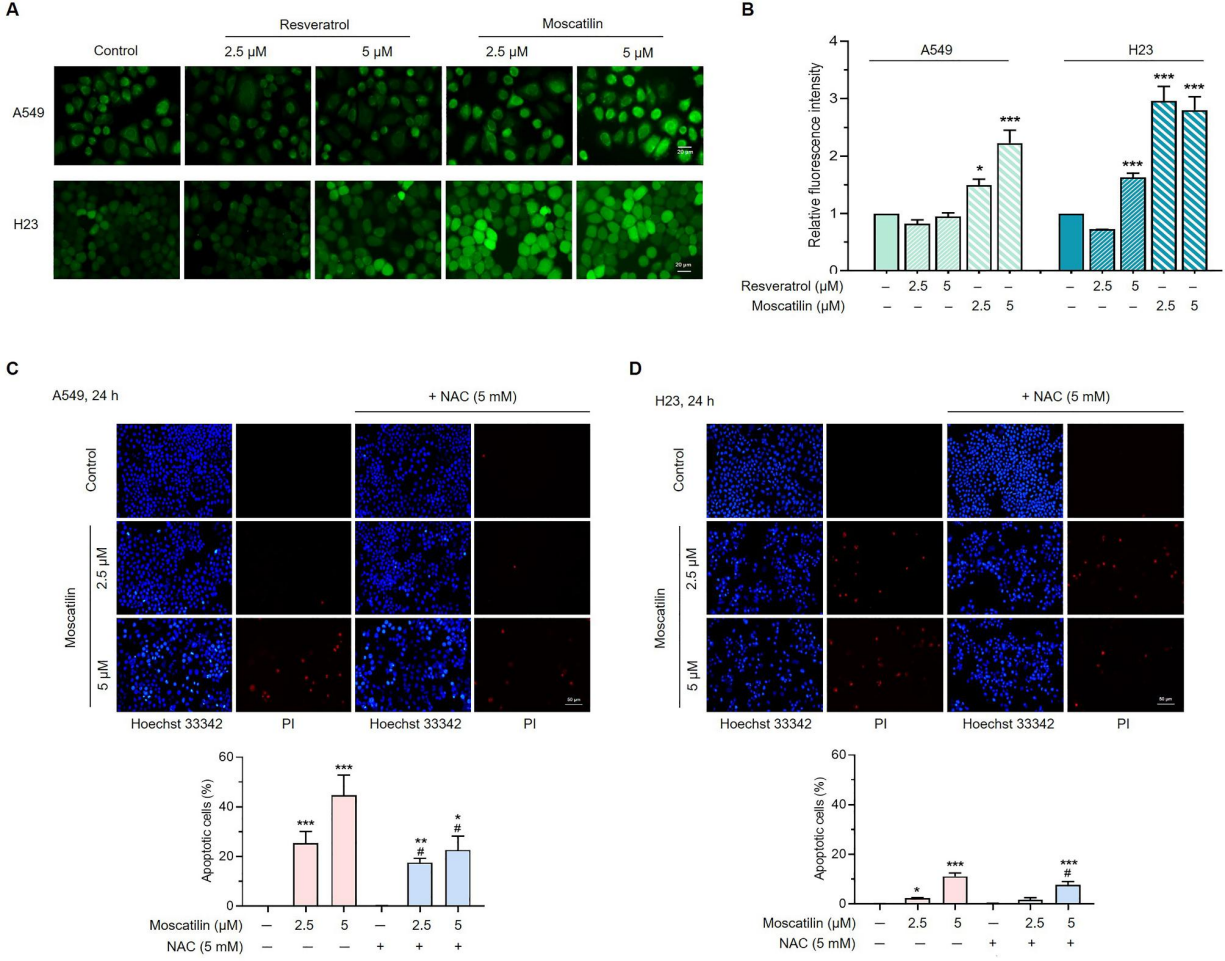
Effects of RES and MOS on intracellular ROS production and MOS induces ROS-mediated apoptosis in lung cancer cells. H23 and A549 were treated with various concentration of RES or MOS at 0, 2.5, and 5 µM. (**A**) ROS levels were detected by fluorescence microscopy using DCFH_2_-DA probe. (**B**) Bar graphs showed the quantitative results of A. (**C**, **D**) H23 and A549 cells were treated with various concentrations of MOS (0, 2.5, and 5 µM) and also pretreated with NAC (5 mM) for 30 min before treating with MOS for 24 h. The apoptotic cells were evaluated using Hoechst 33342/PI double staining. The bottom part shows the quantitative results of C and D. Each value is the mean (± SD) from triplicate experiments. *p < 0.05, **p < 0.01, and ***p < 0.001 vs. control; #*p* < 0.05 vs. MOS at 2.5 or 5 µM.

Oxidative stress induced by the anti-cancer agents may be responsible for cell death [42, 43]. Several recent reports suggested that RES and its derivatives can induce apoptosis in cancer cells via ROS-dependent endoplasmic reticulum stress [44, 45]. We subsequently compared the effect of RES and MOS induced intracellular ROS in lung cancer cell lines (A549 and H23). DCFH_2_-DA fluorescence probe was used to detect intracellular ROS levels in the cells by fluorescence microscopy. Our results showed that relative fluorescence intensity of DCFH_2_-DA was significantly increased in both A549 and H23 cells in a dose-dependent manner (2.5–5 µM) at 2 h of MOS treatment (Fig. 3A and B). For further exploring the possible roles of ROS in MOS-mediated apoptosis of lung cancer cells, NAC (a potent ROS scavenger) was used to pretreat cancer cells for 30 min before treatment with MOS for 24 h. Hoechst 33342/PI double staining revealed that pretreated with NAC reduced the percentage of apoptotic cells induced by treatment with MOS in both A549 and H23 cells (Fig. 3C and D). These results demonstrated that ROS was involved in MOS-induced apoptosis in lung cancer cells.

### Comparative effects of RES and MOS on colony formation and cell cycle arrest in lung cancer cells

**Fig. 4:**
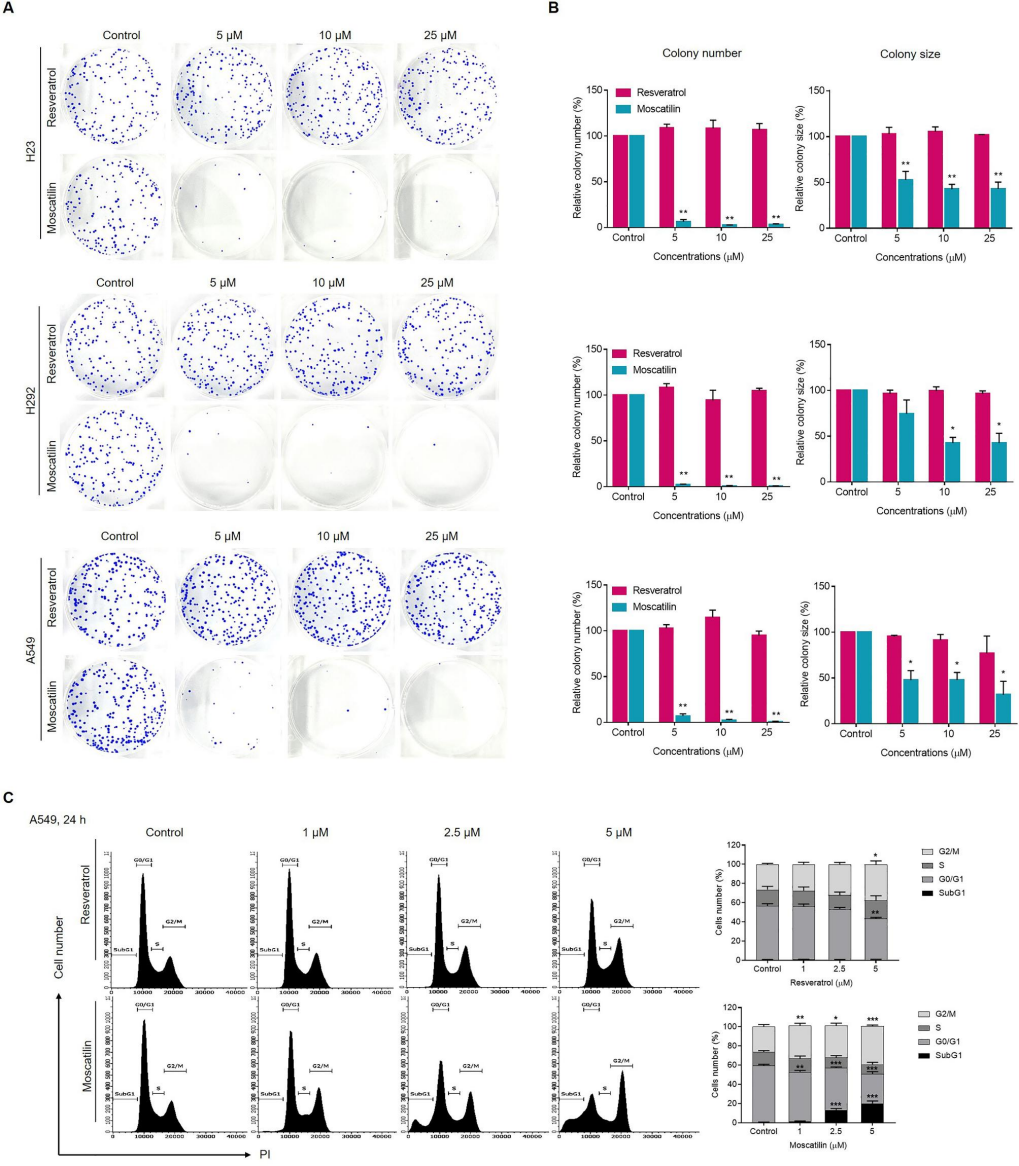
Effects of RES and MOS on colony formation and cell cycle arrest in lung cancer cells. A total of 300 cells were seeded in 6-well plates and then treated with RES or MOS at the concentrations indicated in the figure for 24 h. The colonies were allowed to grow for 7 days and subjected to crystal violet staining. (**A**) Colony formation in lung cancer cell lines following RES or MOS treatment. (**B**) Bar graphs showed the quantitative results of A. The left part shows the colony number and the right part shows colony size. (**C**) After serum starvation of 48 h, cells were treated with RES or MOS, as indicated, for 24 h. DNA content was analyzed by flow cytometer using PI staining. The left part shows the summary of percentages of the cells in sub-G1, G0/G1, S, and G2/M phases. Each value is the mean (± SD) from triplicate experiments. *p < 0.05, **p < 0.01, and ***p < 0.001 vs. control

The colony formation assay was used to determine the effectiveness of RES and MOS in decreasing the growth and proliferation of lung cancer cells. Three lung cancer cell lines, namely, H23, H292, and A549, were treated with RES or MOS at concentrations of 0, 5, 10, and 25 µM for 24 h, the drugs were then discarded, and colonies were allowed to grow for 7 days. As presented in Fig. 4A, MOS strongly inhibited the proliferation of all cell lines in a dose-dependent manner, whereas RES had no effect. Compared with the control group, MOS significantly reduced the number and size of colonies for all lung cancer cell lines (Fig. 4B). In addition, MOS at 5 µM inhibited the colony formation of H23, H292, and A549 cells by 93.6%, 97.8%, and 93.1%, respectively.

Furthermore, we investigated the possibility that the suppression of cell proliferation results from cell cycle disruption. The effect of RES and MOS on cell cycle distribution was evaluated using flow cytometry. As shown in Fig. 4C, the population of A549 cells in the G2/M phase increased significantly after 24 h of MOS treatment in dose-dependent manner (1–5 µM), indicating that MOS promotes lung cancer cell arrest in the G2/M phase. MOS increases the sub-G1 population (apoptotic population), which is accompanied by a corresponding reduction of the cells in the G0/G1 phases of the cell cycle, whereas RES has no effect (Fig. 4C). These results demonstrate that MOS inhibited lung cancer cell proliferation more effectively than RES.

### Comparative effects of RES and MOS suppress stem-cell-like properties of lung cancer cells

**Fig. 5:**
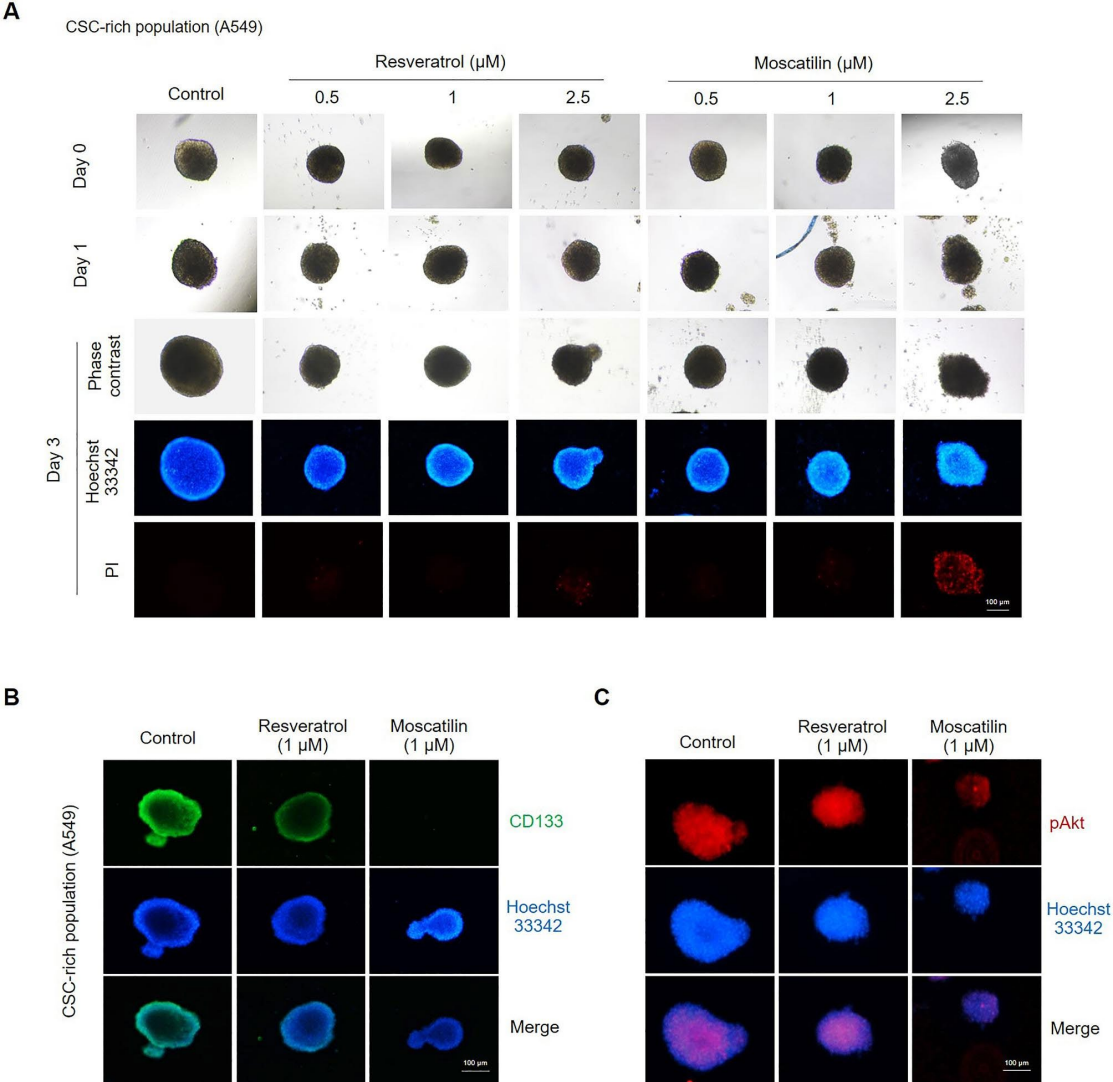
Effects of RES and MOS on suppression of the CSC-like phenotype in lung cancer cells. (**A**) The single spheroid from CSC-rich population of A549 cells were treated with non-toxic concentrations of RES and MOS (0 − 2.5 µM) for 3 days, and CSC viability was determined using Hoechst 33,342/PI double staining. The single spheroid from CSC-rich population of A549 cells were treated with 1 µM of RES or MOS for 24 h. (**B**) The expression levels of CD133 and (**C**) p-Akt were determined using anti-CD133 and anti-p-Akt (Ser473) antibody followed by Alexa Fluor 488-labeled secondary antibody or Alexa Fluor 594-labeled secondary antibody and Hoechst 33,342. The expression was visualized by fluorescence microscopy

To investigate whether RES and MOS suppress stem cell-like properties in lung cancer cells, CSC-rich tumor sphere formation assay was performed to examine whether RES and MOS affect the lung CSCs. CSC-rich populations of A549 cells were generated as described in the *Methods*, and the CSC populations were treated with nontoxic concentrations of RES or MOS (0–2.5 µM) for 3 days. To measure the viability and apoptosis of CSCs, Hoechst 33342/PI double staining was performed on day 3 after treatment with the compounds. The CSC viability was significantly decreased, as indicated by condensed, and fragmented nuclei stained with the DNA dye Hoechst 33342. PI-stained CSC appeared bright red, indicating cell death, and rupture. In the spheroid formation assay, treatment with both RES and MOS reduced the spheroid size and suppressed CSC viability in a dose-dependent manner compared with the untreated control (Fig. 5A).

CD133 protein is a key CSC marker for lung cancer and has been widely used to indicate lung CSCs [46]. CD133-positive cancer cells have self-renewal properties and ability to generate new tumors, whereas CD133-negative cancer cells lacked such potentials [47]. Several studies have implied that certain Akt-dependent pathways are required for the maintenance of CSC proliferation and characteristics [20] a and that CD133 regulates CSC growth and chemoresistance by activating PI3K-AKT signaling [48]. Then, we investigate whether RES and MOS affect CD133 expression and activated Akt (p-Akt). The CSC-rich population was treated with a nontoxic concentration (1 µM) of RES or MOS for 24 h, and the expressions of CD133 and p-Akt were evaluated by immunofluorescence assay. As shown in Fig. 5B, both RES, and MOS reduced CD133 expression in CSCs. MOS exhibited a more potent effect on the suppression of CD133 compared with the parental compound RES. In addition, only MOS could reduce phosphorylated Akt (p-Akt) levels in CSC compared with the control (Fig. 5C). These results suggest that both RES and MOS can suppress the cancer stem-like phenotype of lung CSCs. However, MOS possesses higher potency to suppress CSCs and can inhibit Akt activity.

### Effects of MOS on the inhibition of CSC-like phenotypes through inhibiting akt signaling pathways

**Fig. 6:**
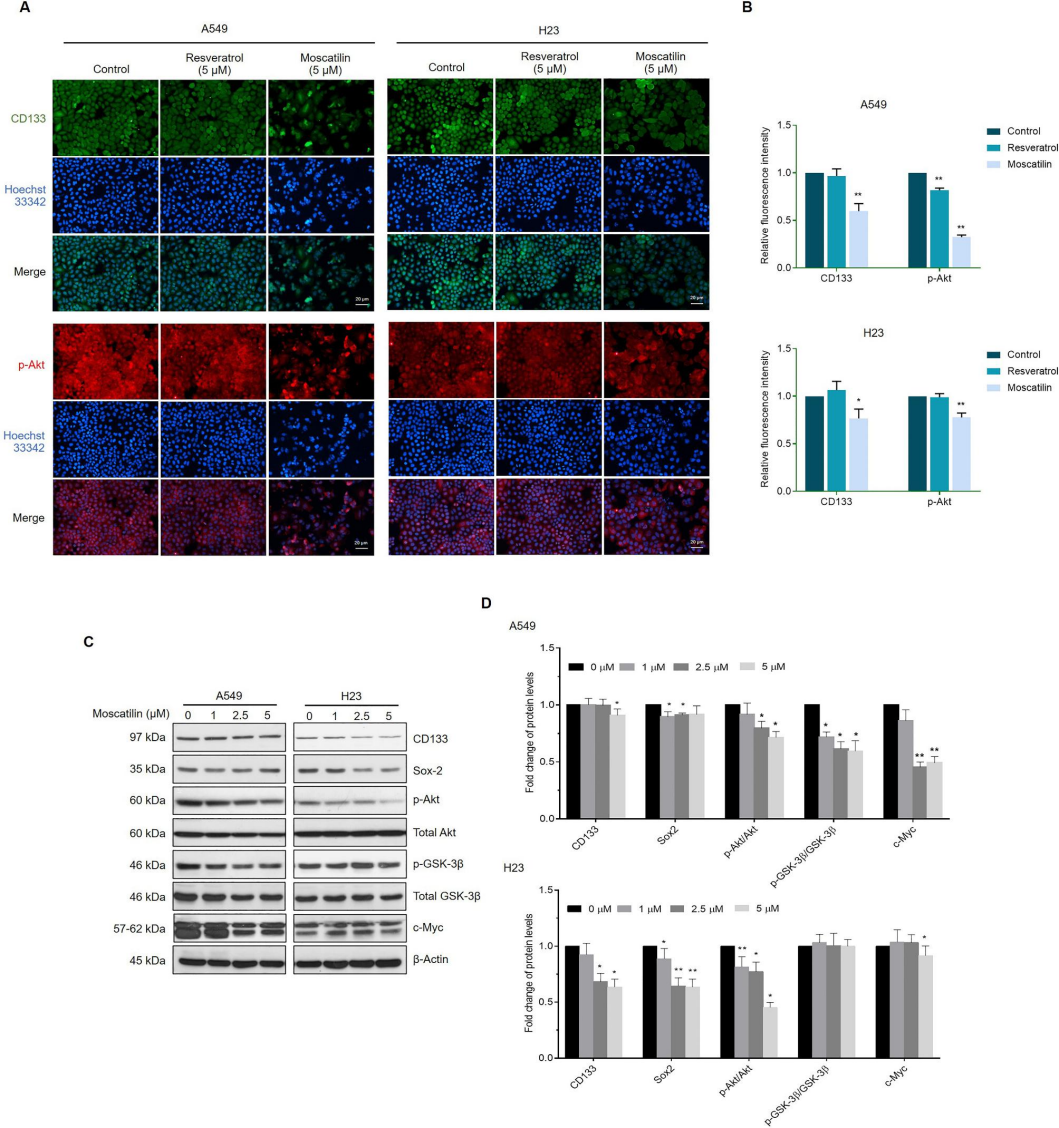
MOS reduces CSC marker and pluripotency transcription factor. (**A**) A549 and H23 cells were treated with 5 µM of RES or MOS for 24 h. The expression levels CD133 and p-Akt were determined using anti-CD133 and anti-p-Akt (Ser473) antibody followed by Alexa Fluor 488-labeled secondary antibody or Alexa Fluor 594-labeled secondary antibody and Hoechst 33342. The expression was visualized by fluorescence microscopy. (**B**) Bar graphs showed the quantitative results of A. (**C**) A549 and H23 cells were treated with MOS (0–5 µM) for 24 h, the levels of CD133, Sox2, p-Akt, total Akt, p-GSK-3β, total GSK-3β, and c-Myc were examined by Western blot analysis. β-actin was utilized as a loading control. The original image of blotting bands was shown in Additional file 1. (**D**) Bar graphs showed the quantitative results of C. Each value is the mean (± SD) from triplicate experiments. *p < 0.05 and **p < 0.01 vs. control

Having shown the effects of RES and MOS on CSC-rich populations, we further confirmed the effect of MOS and RES on cellular signaling that was known to control CSC properties. As shown in Fig. 6A and B, at the same concentration (5 µM), MOS significantly reduced the expression levels of CSC marker CD133 and p-Akt in both A549 and H23 cells, whereas RES can reduce only p-Akt levels in A549 cells. These results suggest that (i) MOS has more potency to suppress CSC than RES when used at the same concentration, and (ii) MOS suppressed CSC-like phenotypes at least in part by inhibiting Akt signaling.

Then, we evaluated the effect of MOS on CSC-like phenotypes involved in the Akt signaling pathway. To determine the underlying mechanism of MOS suppression of CSC-like phenotypes, A549 and H23 cells were treated with MOS (0–5 µM) for 24 h, and the expression levels of CD133, Sox2, p-Akt, Akt, p-GSK-3β, and GSK-3β were determined by Western blot analysis. MOS significantly reduced the expression levels of p-Akt/Akt, p-GSK-3β/GSK-3β, and c-Myc in a dose-dependent manner in A549 cell; however, the p-GSK-3β/GSK-3β ratio was not altered in H23 cells (Fig. 6C and D). In addition, CD133 and pluripotent transcription factors Sox2 were significantly reduced following the decrease in p-Akt/Akt in H23 and A549 cells. Significant suppression of CD133 and Sox2 was first observed with 2.5 µM of MOS in H23 (Fig. 6C and D).

**Fig. 7:**
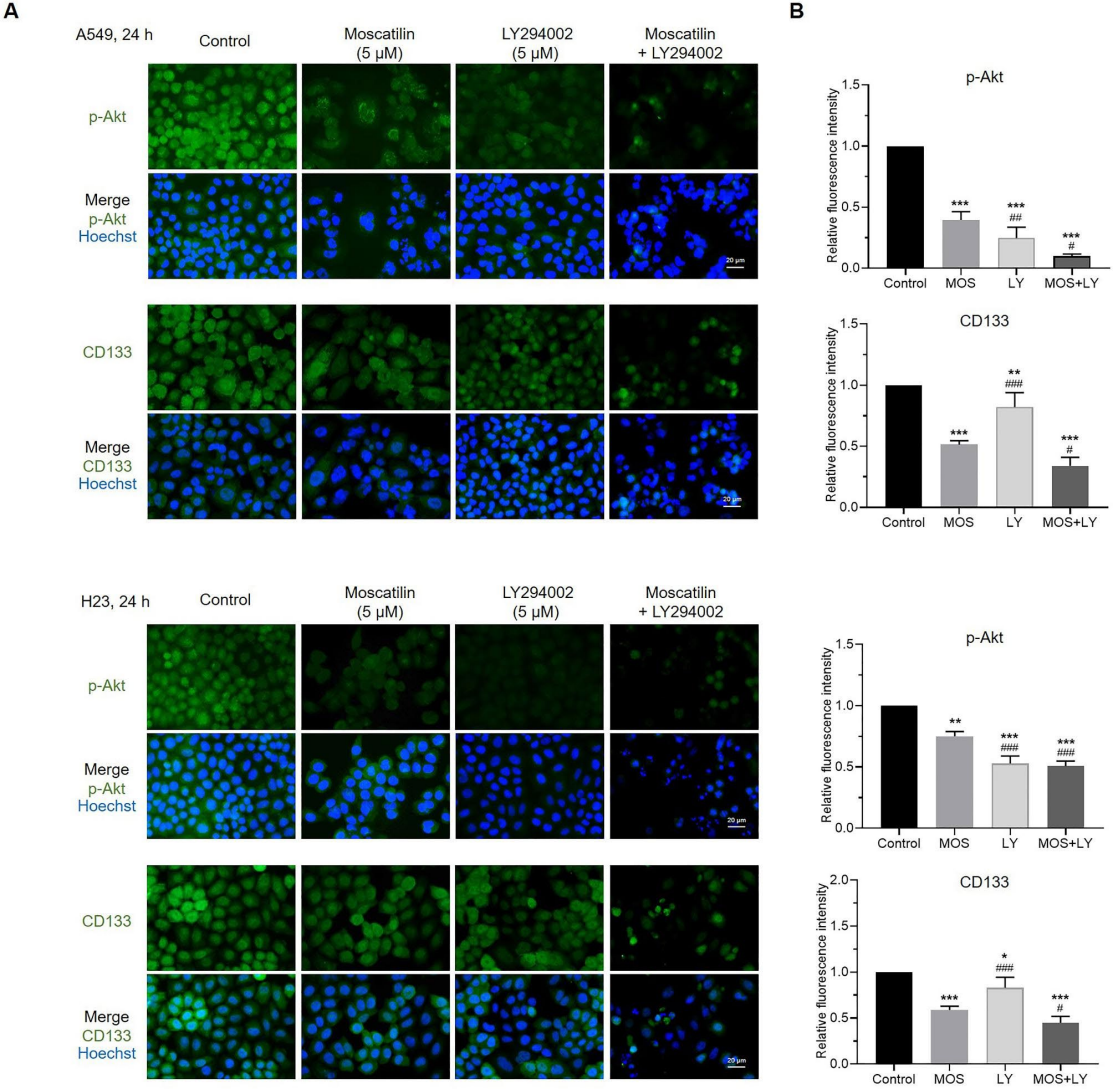
MOS suppresses CSC-like phenotypes by inhibiting Akt signaling pathways. H23 and A549 cells were treated with 5 µM of MOS and also pretreated with LY294002 (5 µM) for 30 min before treating with MOS for 24 h. (**A**) The expression levels of CD133 and p-Akt were determined using anti-CD133 and anti-p-Akt (Ser473) antibody followed by Alexa Fluor 488-labeled secondary antibody and Hoechst 33342. The expression was visualized by fluorescence microscopy. (**B**) Bar graphs showed the quantitative results of A. Each value is the mean (± SD) from triplicate experiments. *p < 0.05, **p < 0.01, and ***p < 0.001 vs. control; #*p* < 0.05, ##*p* < 0.01, and ###*p* < 0.001 vs. MOS at 5 µM

We also confirmed the effect of MOS suppressed CSC-like phenotype via Akt signaling compared to PI3K/Akt pathway inhibitor LY294002. Inactivation of PI3K using LY294002 has been demonstrated to suppress the Akt activity through inhibiting the phosphorylation of Akt at both T308 and S473, consequently inducing cell cycle arrest and apoptosis [49, 50]. To investigate whether the effect of MOS on p-Akt and CD133 protein levels was similar to that of LY294002. A549 and H23 cells were treated with MOS, LY294002, or the combination at the same concentration (5 µM) for 24 h. Fluorescence analysis revealed that both MOS and LY294002 suppressed p-Akt and CD133, while it was more strongly inhibited by the MOS/LY294002 combination (Fig. 7A and B). Thus, MOS mediated inhibition of CSC-like phenotypes in lung cancer cells via Akt signaling.

### Molecular docking and MD simulations

**Fig. 8:**
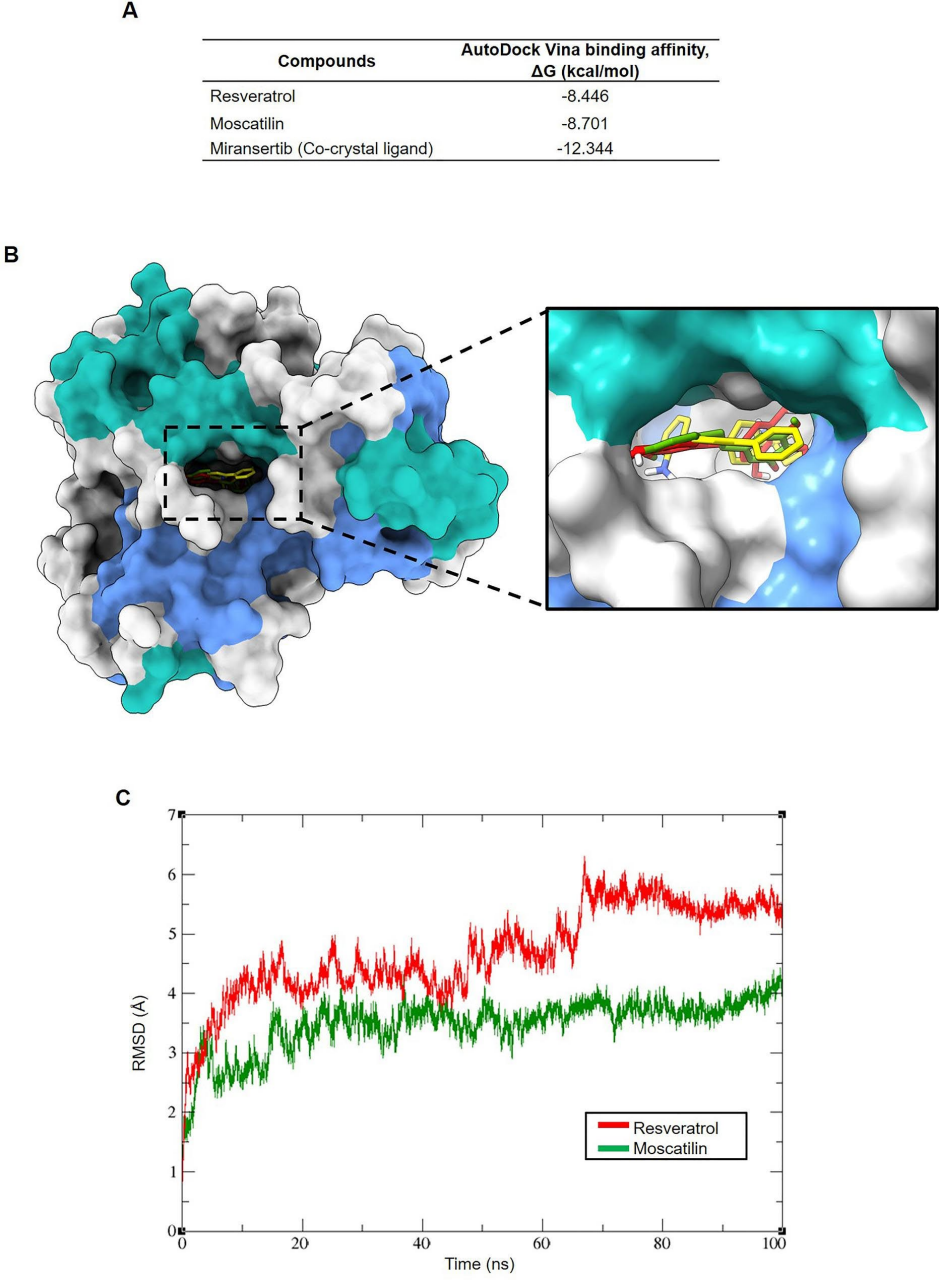
Molecular docking and MD simulation of RES and MOS with Akt. (**A**) Binding affinities of compounds docked with the allosteric site of Akt1 (**B**) The superimposed docking poses of RES (red), moscatillin (green), and minransertib (yellow). (**C**) RMSD values of the backbone atoms of Akt1/ligand complexes

To predict the possible binding mode of RES and MOS with Akt, a molecular docking study was performed. RES and MOS were docked into the allosteric binding site of Akt1 using AutoDock Vina. The values of the binding affinities of compounds are presented in Fig. 8A. As presented in the visualization of the compound’s binding poses (Fig. 8B), as expected, the compounds are well-contained within the allosteric binding site of Akt1. Based on the predicted binding affinities and binding pose, the compounds were further selected for MD simulations using AMBER18.

A 100-ns MD simulation of each Akt1/ligand complex was conducted to optimize the docking-predicted binding modes of two natural compounds. To explore the dynamic stability of the two systems, the root mean square deviation (RMSD) values were calculated during 100-ns MD simulations. As shown in Fig. 8C, the RMSD plot indicated that all two systems reached equilibrium after 70 ns. Based on the stable MD trajectory, MM/GBSA binding free energy was calculated to evaluate the binding affinities of two natural compounds to Akt1. As summarized in Table 1, compounds RES and MOS had − 17.0134 and − 32.8245 kcal/mol to Akt1, respectively. The MOS complex had the highest binding free energy, which was attributed to the dominant contribution of the van der Waals forces. In comparison, RES had weakened binding affinities, which were mainly associated with decreased van der Waals forces. However, electrostatic energy plays a particular role in all systems. A strong influence was observed from the gas-phase free energy when assessed against it, with minor contributions from the solvation-free energy. As a result, the hydrophobic contact may be crucial to the efficient binding relationship between ligands and the allosteric site of Akt1.

**Table 1:** Binding free energies of the Akt1 and natural compound complexes

Compounds	VDW	ELE	EGB	ESURF	ΔG gas	ΔG solv	ΔTOTAL
RES	−29.6305	−8.9856	26.1967	−4.5940	−38.6161	21.6027	−17.0134
MOS	−41.6353	−15.9726	30.4154	−5.6320	−57.6079	24.7834	−32.8245

All units are given in kcal/mol. Abbreviations: VDW, the van der Waals forces; ELE, the electrostatic energy; EGB, the solvation free energy; ESURF, the nonpolar contribution to the solvation free energy; ΔG gas, the gas-phase free energy; ΔG solv, the solvation free energy; ΔTOTAL, the total free energy.

**Fig. 9:**
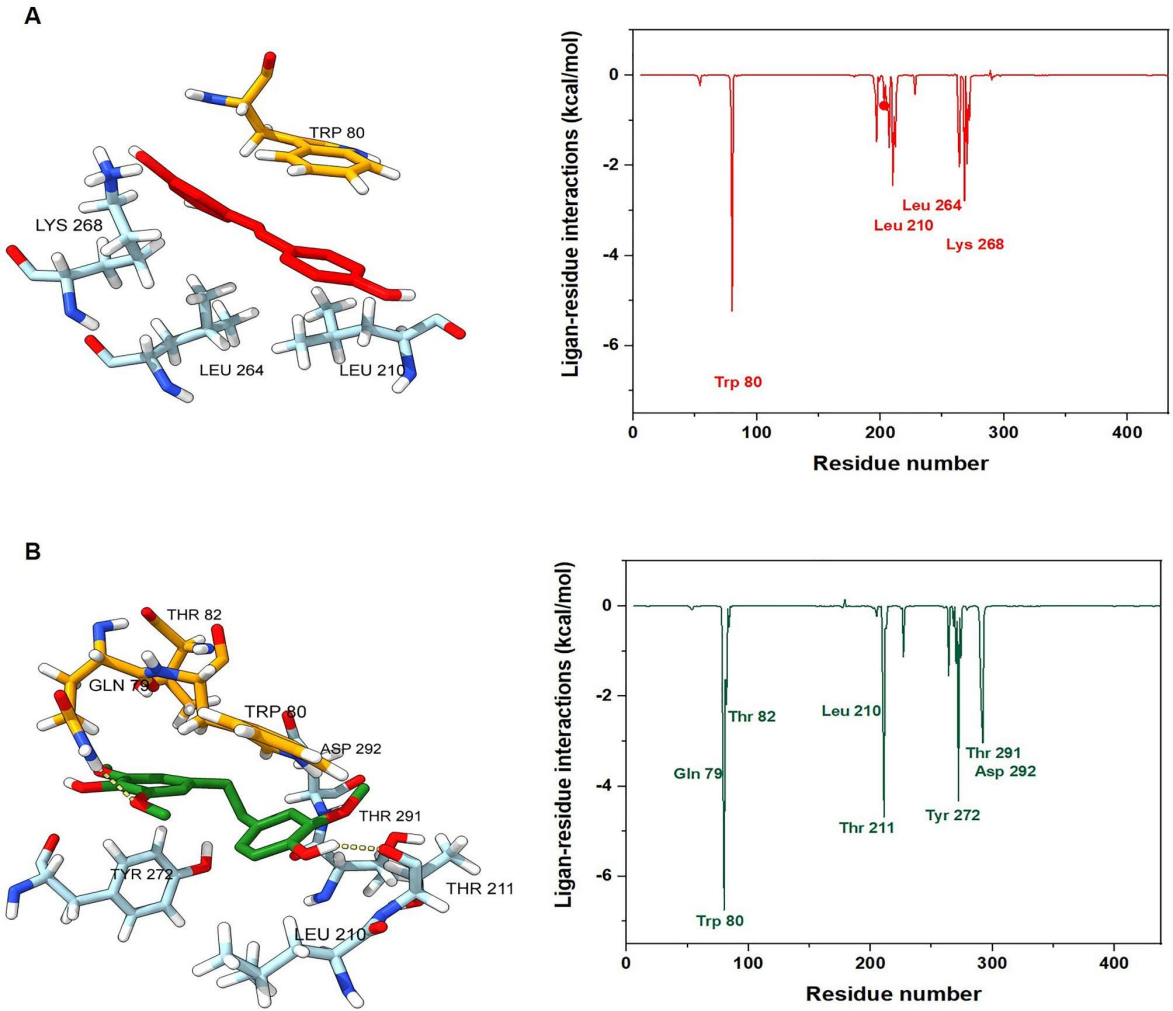
MM/GBSA per-residue binding energy decomposition of (**A**) RES and (**B**) MOS in complex with Akt1

The per-residue binding energy decomposition was performed to obtain details of Akt1–ligand complexes and identify the key residues responsible for ligand binding. Generally, the residue would be considered necessary for binding when the binding energy is more negative than − 2 kcal/mol. Trp80 and Tyr272 have been reported as essential residues for the potent allosteric Akt-1 inhibitor in clinical trials [25, 51]. As shown in Fig. 9, the MD binding pose shows that MOS is the most promising Akt1 inhibitor. MOS formed two hydrogen bonds with Gln79 and Thr211 and hydrophobic interactions with Trp80 and Thr82 in the PH domain and Leu210, Tyr 272, Thr291, and Asp292 in the kinase domain (Fig. 9B). These results suggest that MOS might inhibit Akt1 via an allosteric mechanism.

## Discussion

CSCs in cancer were found to resist chemotherapeutic drugs and induce disease recurrence after therapy [52]. CSCs can regenerate tumors through self-renewal and differentiation to create heterogeneous tumor cells [53]. In this study, we utilized RES, a well-known natural product-derived compound with anticancer activity as the lead compound for the CSC-targeting activity. The modification of the chemical structure of RES to MOS was found to enhance CSC-specific suppression through Akt inhibition. From our results, MOS was more potent in suppressing cell viability and colony formation and inducing apoptosis of lung cancer cells than RES (Figs. 2 and 4). MOS can suppress cell viability in lung cancer cell lines A549 and H292 with IC_50_ of less than 10 µM, implying that MOS might be a promising anticancer agent for the treatment of lung cancer. In addition, MOS exhibits low toxicity to normal cells (Fig. 2A and B), indicating a positive therapeutic index. Colony formation assay was performed to detect the inhibitory effect of RES and MOS on the proliferative ability. The assay is currently widely used to examine the effect of agents with potential clinical applications [54], and it demonstrates the capacity of cancer cells to generate viable colonies following drug treatment. Interestingly, H23 appears to be less sensitive to MOS with an IC_50_ of > 30 µM, however, at low doses of MOS (5 µM) can suppress colony formation by more than 90%, similar to other lung cancer cell lines A549 and H292 (Fig. 4A and B). Cell cycle regulation plays a crucial role in lung cancer cell growth and survival. We demonstrated that MOS triggered G2/M phase arrest at a low concentration (1 µM), resulting in reduced cell proliferation and apoptosis in a dose-dependent manner in lung cancer cells (Figs. 2 and 4). Our findings are consistent with studies on colorectal [55] and esophageal [56] cancer cell in which MOS activated cell apoptosis by inducing G2/M arrest. These outcomes inferred that MOS might be qualified for both lung cancer treatment and prevention.

Several studies have demonstrated that various anti-cancer agents induce cancer cell apoptosis through ROS generation [57]. It has been reported that NAC treatment effectively reduced the increase in ROS level of pancreatic cancer cells Panc-1 caused by MOS, as well as inhibited the ROS-mediated activation of the JNK/SAPK signaling pathway and induced apoptosis [58]. The present study revealed that treatment with MOS led to an increase in ROS production, and as expected, NAC can reduce MOS-induced cell apoptosis in lung cancer cells A549 and H23 (Fig. 3C and D). These results indicate that the anticancer activity of MOS was caused by increasing ROS generation.

MOS was demonstrated to have a superior activity to suppress CSCs than RES at the same concentration, indicated by the reduction of CSC marker, pluripotent transcription factor, and stem cell-like phenotypes (Figs. 5 and 6). Lung cancer is a CSC-containing cancer, and studies utilized specific protein markers and spheroid formation behavior for CSC detection [59]. In this study, we used the CSC marker CD133 as it was demonstrated to be suitable for CSC detection in lung cancer [60]. In terms of the upstream signaling pathway, Akt is an important protein in regulating CSC [20]. Interestingly, CD133 interacted with the PI3K–AKT pathway. Wei et al. showed that the PI3K/Akt signaling pathway can be activated by CD133/p85 interaction [61]. The CD133/Akt signal was found to be associated with the increase in tumorigenicity. The CD133-positive cancer cells also had a higher level of activated Akt than CD133-negative cancer cells [61]. Moreover, we found a concomitant decrease in CD133 and phosphorylated Akt in response to MOS treatment in both adherent (monolayer) and CSC-rich spheres (Figs. 5 and 6A, and 6B). Our results show that the expression levels of p-Akt and Sox2 significantly decreased in response to MOS treatment (Fig. 6C and D). Consistent with our findings, the inhibition of Akt signaling suppresses the self-renewal ability and expansion of CSC together with Sox2 reduction [62].

GSK-3β was recognized as a downstream effector of Akt and has been indicated as a cellular regulator of growth, drug resistance, metastasis, and CSC maintenance [63]. Akt can phosphorylate GSK-3β at Ser9, resulting in its deactivation [64]. Several transcription factors, including c-Myc, are regulated by GSK-3β activity. c-Myc functions as a proto-oncoprotein transcription factor that activates the expression of genes controlling cell division, differentiation, and maintenance of stem characteristics [22, 65]. GSK-3β regulates c-Myc via phosphorylation at Thr58, resulting in ubiquitin-dependent degradation of c-Myc [66]. The expression levels of p-Akt, p-GSK-3β (Ser9)/GSK-3β, and c-Myc proteins significantly decreased in response to MOS (Fig. 6C and D), suggesting that MOS may suppress lung CSCs through the Akt/GSK-3β/c-Myc pathway. We also confirmed that combination of MOS with the Akt inhibitor LY294002 more effectively suppresses Akt activity (p-Akt) and expression of stemness markers CD133 compared to MOS or LY294002 treatment, demonstrating that MOS could suppress CSC-like phenotypes partly through downregulation of p-Akt.

Compounds possessing the ability to inhibit Akt are under investigation in a clinical setting [67]. The majority of drug actions for Akt inhibition are through interaction with the ATP-binding site or binding to the allosteric site of Akt [51]. To confirm our hypothesis that RES or MOS might be potential Akt inhibitors, we performed molecular docking, and MD simulation. The results revealed that MOS acted as a potential allosteric inhibitor of Akt rather than RES. The binding affinity of MOS with Akt was − 8.70 kcal/mol, the binding free energy of MM/GBSA was − 32.8245 kcal/mol, and MOS can interact with Trp80 and Tyr272, which were reported as essential residues for the potent allosteric Akt1 inhibitor in clinical trials. These results demonstrate that MOS can bind to the Akt protein through an allosteric mechanism.

## Conclusion

In this study, we have unraveled the anticancer activity of RES and its modified analog MOS against CSC of lung cancer cells. MOS was more potent than its parental compound in terms of cytotoxicity and CSC suppression. This study demonstrated that MOS suppresses lung CSCs via the Akt/GSK-3β/c-Myc pathway. Furthermore, molecular docking, and MD simulation revealed the possibility that MOS interacts with the Akt molecule. The binding affinity to the allosteric site of the Akt protein of MOS was greater than that of RES. Our findings highlight the therapeutic potential of MOS in the treatment of lung CSCs and support further research toward the clinical development of this compound.

## Electronic supplementary material

Below is the link to the electronic supplementary material.

Additional file 1

## Data Availability

The datasets used and/or analyzed during the current study are available from the corresponding author on reasonable request.

## Abbreviations

Akt: Protein kinase B (PKB) or serine/threonine-specific protein kinase
CSC: Cancer stem-like cell
GSK-3β: Glycogen synthase kinase 3β
MOS: Moscatilin
NAC: *N-*acetyl-L-cysteine
RES: Resveratrol
ROS: Reactive oxygen species
Sox2: Sex-determining region Y-box 2
