# Supplementary figures and images for: Structural modification of resveratrol analogue exhibits anticancer activity against lung cancer stem cells via suppression of Akt signaling pathway
Source: BMC Complement Med Ther. 2023 Jun 3;23:183. doi: 10.1186/s12906-023-04016-6 (PMC10239606; doi:10.1186/s12906-023-04016-6)

Figure 6 Supplemental C

A549 cells

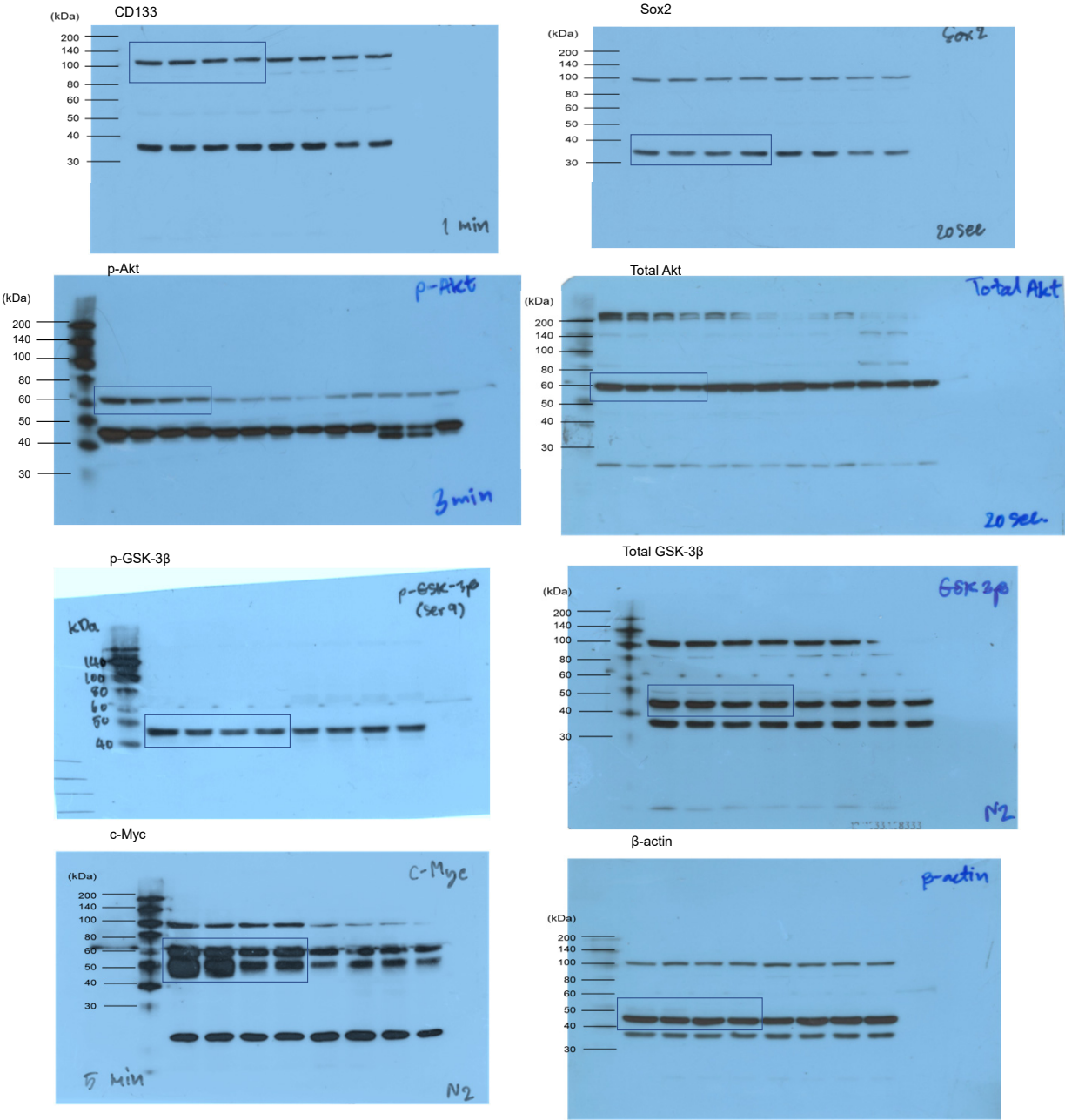

H23 cells

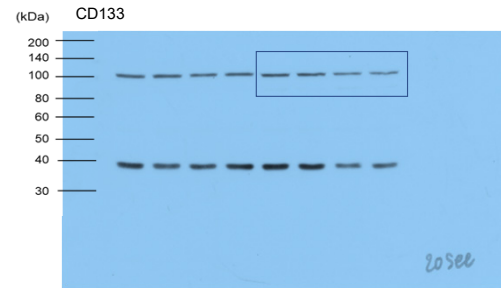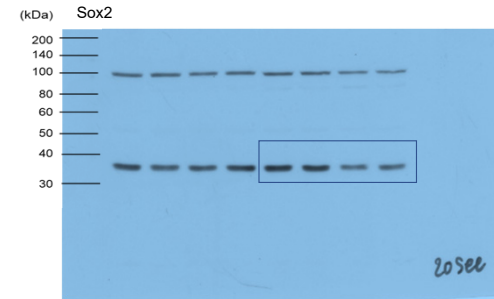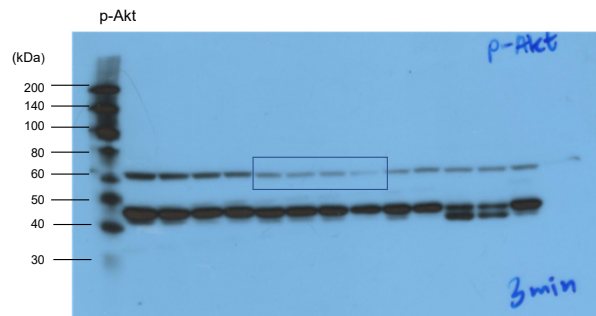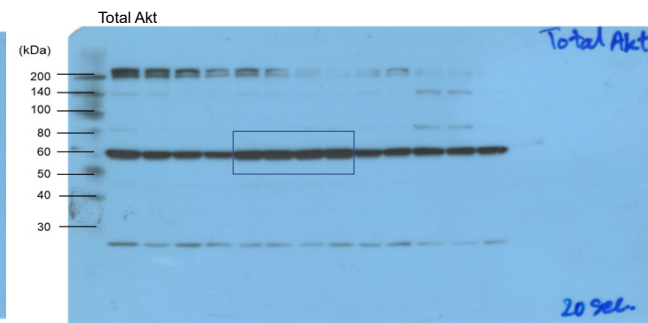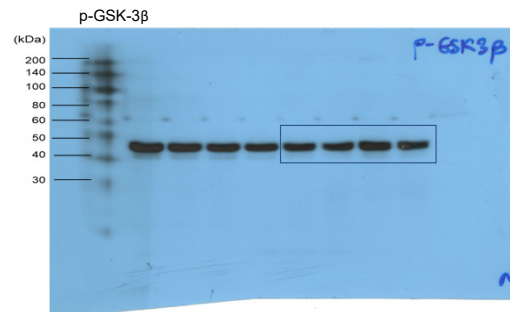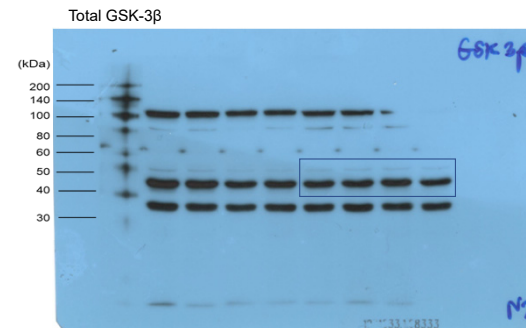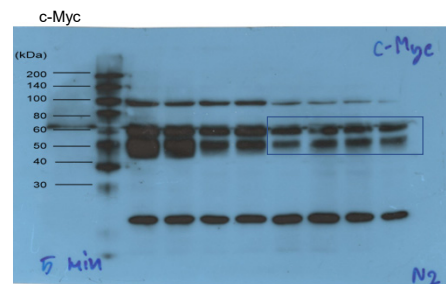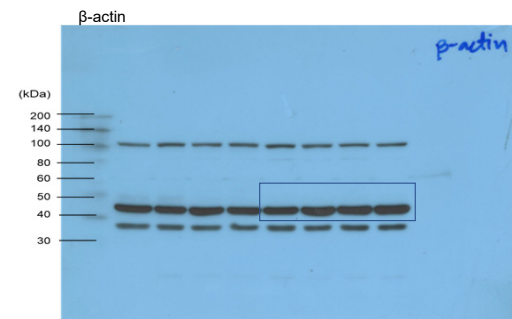

Supplement: Supplementary file 1 — Additional file 1 [file 12906_2023_4016_MOESM1_ESM.pdf]
